# How to write a successful grant application: guidance provided by the European Society of Clinical Pharmacy

**DOI:** 10.1007/s11096-023-01543-7

**Published:** 2023-03-06

**Authors:** Anita E. Weidmann, Cathal A. Cadogan, Daniela Fialová, Ankie Hazen, Martin Henman, Monika Lutters, Betul Okuyan, Vibhu Paudyal, Francesca Wirth

**Affiliations:** 1 Department of Clinical Pharmacy, Innsbruck University, Innsbruck, Austria; 2 School of Pharmacy and Pharmaceutical Sciences, Trinity College Dublin, Dublin, Ireland; 3 Department of Social and Clinical Pharmacy, Faculty of Pharmacy in Hradec Králové, Charles University, Hradec Králové, Czech Republic; 4 Department of Geriatrics and Gerontology, 1st Faculty of Medicine, Charles University, Prague, Czech Republic; 5 Julius Centre for Health Sciences and Primary Care, University Medical Center Utrecht, Utrecht, The Netherlands; 6 Trinity College Dublin, Dublin, Ireland; 7 Kantonsspital Aarau, Aarau, Switzerland; 8 Department of Clinical Pharmacy, Faculty of Pharmacy, Marmara University, Istanbul, Turkey; 9 University of Birmingham, Birmingham, United Kingdom; 10 Department of Pharmacy, University of Malta, Msida, Malta

**Keywords:** Clinical pharmacy, Economics, Funding, Grants, Peer review, Writing

## Abstract

Considering a rejection rate of 80–90%, the preparation of a research grant is often considered a daunting task since it is resource intensive and there is no guarantee of success, even for seasoned researchers. This commentary provides a summary of the key points a researcher needs to consider when writing a research grant proposal, outlining: (1) how to conceptualise the research idea; (2) how to find the right funding call; (3) the importance of planning; (4) how to write; (5) what to write, and (6) key questions for reflection during preparation. It attempts to explain the difficulties associated with finding calls in clinical pharmacy and advanced pharmacy practice, and how to overcome them. The commentary aims to assist all pharmacy practice and health services research colleagues new to the grant application process, as well as experienced researchers striving to improve their grant review scores. The guidance in this paper is part of ESCP’s commitment to stimulate “*innovative and high-quality research in all areas of clinical pharmacy*”.

## Overview

Writing research grants is a central part of any good quality research. Once a detailed research proposal has been submitted, it is subjected to an expert peer review process. Such reviews are designed to reach a funding decision, with feedback provided to improve the study for this and any future submissions. Depending on the length of the proposal, complexity of the research and experience of the research team, a proposal can take between six to twelve months to write [1]. Ample time must be given to the writing of hypothesis/research aim, budgeting, discussion with colleagues and several rounds of feedback [2]. The draft research proposal should always be completed well before the deadline to allow for last minute delays. An application which is not fully developed should not be submitted since it will most likely be rejected [3].

Despite the large effort that goes into each grant application, success rates are low. Application success rates for Horizon 2020 were < 15% [4] and < 20% for the National Institute of Health (NIH) [5–8]. With these statistics in mind, it is evident that often repeated submissions are required before securing funding. Due to a paucity of specific clinical pharmacy grant awarding bodies, writing a grant application for a clinical pharmacy or pharmacy practice research project often involves multidisciplinary collaborations with other healthcare professions and focus on a specific patient population or condition. There is no guarantee of success when trying to secure funding for research. Even the most seasoned researchers will have applications rejected. The key is to never give up. This commentary provides useful pointers for the planning and execution of grant writing.

## Conceptualising your research idea

Before writing a research grant proposal/application, consider what the research should achieve in the short, medium, and long term, and how the research goals will serve patients, science and society [9, 10]. Practical implications of research, policy impact or positive impact on society and active patient/public involvement are highly valued by many research agencies as research should not be conducted “only for research”, serving the researchers’ interests. EU health policy and action strategies (CORDIS database) and other national strategies, such as national mental health strategy for grants within mental disorders, should be considered, as well as dissemination strategies, project deliverables, outcomes and lay public invitations to participate. The Science Community COMPASS has developed a useful “Message Box Tool” that can help in the identification of benefits and solutions, as well as the all-important “So What?” of the research [11]. Clearly determine what the lead researcher’s personal and professional strengths, expertise and past experiences are, and carefully select the research team to close these gaps [12–14].

## How to find the right funding call

When trying to identify the right type of grant according to the research ambitions, one should be mindful that several types of grants exist, including small project grants (for equipment, imaging costs), personal fellowships (for salary costs, sometimes including project costs), project grants (for a combination of salary and project costs), programme grants (for comprehensive project costs and salary for several staff members), start-up grants and travel grants [15]. Types of grants include EU grants (e.g. Horizon, Norway Grant), commercial grants (e.g. healthcare agencies and insurance companies), New Health Program grants ideal for new, reimbursed clinical pharmacy service projects and national grants (e.g. FWF (Austria), ARRS (Slovenia), NKFIH (Hungary), NCN (Poland), FWO (Belgium), HRZZ (Croatia), GAČR (Czech Republic), SNSF (Switzerland), SSF (Sweden). It is worth remembering that early career researchers, normally within ten years of finishing a PhD, have a particular sub-category within most grants.

Many national agencies only have one “Pharmacy” category. This results in clinical pharmacy and advanced clinical pharmacy practice projects competing with pharmaceutical chemistry, pharmaceutical biology and pharmacy technology submissions, thereby reducing the success rate as these research areas can often be very advanced in most EU countries compared to clinical and advanced pharmacy practice. A second possible submission category is “Public Health”. Several essential factors can impact the grant selection, such as research field, budget capacity, leading researcher’s experience and bilateral grants. Examples of successful clinical pharmacy funded research studies can be found in the published literature [16–20].

## Plan, plan, plan

One key element of successful grant writing is the ability to plan and organise time. In order to develop a realistic work plan and achieve milestones, it is imperative to note deadlines and to be well-informed about the details of what is required. The development of a table or Gantt Chart that notes milestones, outcomes and deliverables is useful [21].

All funders are quite specific about what they will and will not fund. Research your potential funders well in advance. It is vital to pay attention to the aims, ambitions and guidelines of the grant awarding bodies and focus your proposal accordingly. Submitting an application which does not adhere to the guidelines may lead to very early rejection. It is helpful to prepare the grant application in such a way that the reviewers can easily find the information they are looking for [15, 22]. This includes checking the reviewers’ reports and adding “bolded” sentences into the application to allow immediate emphasis. Reviewers’ reports are often available on the agencies’ websites. It is extremely useful to read previously submitted and funded or rejected proposals to further help in the identification of what is required in each application. Most funding agencies publish a funded project list, and the ‘Centre for Open Science (COS) Database of Funded Research’ enables tracking of funding histories from leading agencies around the world [23]. Another useful recommendation is to talk to colleagues who have been successful when applying to that particular funder. Funding agency grant officers can provide advice on the suitability of the proposal and the application process.

It is important to pay particular attention to deadlines for the grant proposal and ensure that sufficient time is allocated for completion of all parts of the application, particularly those that are not fully within one’s own control, for example, gathering any required signatures/approvals. Funders will generally not review an application submitted beyond the deadline.

Lastly, it is important to obtain insight into the decision process of grants. Research applications are sent to several reviewers, who are either volunteers or receive a small compensation to judge the application on previously determined criteria. While the judging criteria may vary from funder to funder, the key considerations are:

1. Is there a clear statement of the research aim(s)/research question(s)/research objective(s)?
2. Is the proposed research “state-of-the-art” in its field and has all relevant literature been reviewed?
3. Is the method likely to yield valid, reliable, trustworthy data to answer question 1.?
4. If the answer to the second question is ’yes’, then what is the impact of financing this study on patient care, professional practice, society etc.?
5. Is there sufficient confidence that the research team will deliver this study on time with expected quality outputs and on budget?
6. Does the study provide value for money?

## How to write

The key to good grant proposal writing is to be concise yet engaging. The use of colour and modern web-based tools such as #hashtags, webpage links, and links to YouTube presentations are becoming increasingly popular to improve the interest of a submission and facilitate a swift decision-making process. Ensure use of the exact section headings provided in the guidance, and use the keywords provided in the funding call documentation to reflect alignment with the funding bodies’ key interests. Attention to detail cannot be overstated; the quality and accuracy of the research proposal reflect the quality and accuracy of the research [24]. Try to adopt a clear, succinct, and simple writing style, making the grant easy to read. Having a clear focus can help to boost a grant to the top of a reviewer’s pile [25, 26]. A clearly stated scientific question, hypothesis, and rationale are imperative. The reviewer should not have to work to understand the project [27]. Allow for plenty of time to incorporate feedback from trusted individuals with the appropriate expertise and consider having reviews for readability by non-experts.

## What to write

### Abstract, lay summary and background/rationale

Take sufficient time to draft the scientific abstract and summary for the lay public. These should clearly state the long-term goal of the research, the aim and specific testable objectives, as well as the potential impact of the work. The research aim is a broad statement of research intent that sets out what the project hopes to achieve at the end. Research objectives are specific statements that define measurable outcomes of the project [28, 29].

The lay summary is important for non-subject experts to quickly grasp the purpose and aims of the research. This is important in light of the increased emphasis on patient and public involvement in the design of the research. The abstract is often given little attention by the applicants, yet is essential. If reviewers have many applications to read, they may form a quick judgement when reading the abstract. The background should develop the argument for the study. It should flow and highlight the relevant literature and policy or society needs statements which support the argument, but at the same time must be balanced. It should focus on the need for the study at the local, national and international level, highlighting the knowledge gap the study addresses and what the proposed research adds. Ensure this section is well-referenced. The innovation section addresses the ‘‘So what?’’ question and should clearly explain how this research is important to develop an understanding in this field of practice and its potential impact. Will it change practice, or will it change the understanding of the disease process or its treatment? Will it generate new avenues for future scientific study? [30].

### Hypothesis/aims and objectives

For the hypothesis, state the core idea of the grant in one or two sentences. It should be concise, and lead to testable specific aims. This section is fundamental; if it is unclear or poorly written, the reviewers may stop reading and reject the application. Do not attempt to make the aims overly complex. Well-written aims should be simply stated. Criteria such as PICO (population, intervention, comparison, outcomes) [31], and FINER (feasible, interesting, novel, ethical, relevant) [32], provide useful frameworks to help in writing aim(s), research question(s), objective(s) and hypotheses. Pay attention to the distinction between aim(s), research question(s), objective(s) and hypotheses. While it is tempting to want to claim that enormously complex problems can be solved in a single project, do not overreach. It is important to be realistic [25].

### Experimental design, methods and expertise

The methodology is one of the most important parts of getting a grant proposal accepted. The reviewing board should be convinced that the relevant methodology is well within the research teams’ expertise. Any evidence of potential success, such as preliminary results or pilot studies strengthen the application significantly [33]. The methodology must relate directly to the aim. Structuring this section into specific activities/ set of activities that address each research question or objective should be considered. This clarifies how each question/ objective will be addressed. Each work-package should clearly define the title of the research question/objective to be addressed, the activities to be carried out including milestones and deliverables, and the overall duration of the proposed work-package. Deliverables should be presented in table format for ease of review. Each subsequent work-package should start once the previous one has been completed to provide a clear picture of timelines, milestones and deliverables which reflect stakeholder involvement and overall organisation of the proposed project. Using relevant EQUATOR Network reporting guidelines enhances the quality of detail included in the design [34]. Key elements of this methodology are detailed in Table 1.

**Table 1:** Summary of the key elements of the experimental design, methods and expertise

Key elements of experimental design, methods and expertise	
Study design	State, justify and explain the study design and methodology.
Setting	Where will the study be conducted? Explain and justify the setting.
Target population	What is the study population? What are the inclusion and exclusion criteria?
Sampling, sample size	Is sampling required? If so, what is the sampling approach and sample size needed?
Recruitment	What is the approach for recruitment?
Data collection	What is the plan for data collection? How are tools to be developed, tested and piloted?
Outcome measures	What is going to be ‘measured’ (noting that the term ‘measure’ is different in qualitative studies)? The outcome measures should directly relate to the specific research questions/ objectives.
Validity, reliability, trustworthiness	What steps are planned to maximise data validity and reliability (and possibly responsiveness) for quantitative studies and trustworthiness for qualitative studies?
Analysis	What are the plans for analysis? The analysis plan must relate directly to the research question (s)/ objective (s).
Monitoring	What are the milestones and key performance indicators for the study? Depending on the funding body and the nature of the study, a monitoring and oversight/ advisory committee may need to be established.
Limitations, mitigation	What are the risks? What could go wrong? It is imperative to highlight these and plan mitigation measures.
Expertise	The research team must have the appropriate level of experience and expertise from relevant disciplines to give the reviewers confidence that the study will be delivered as planned. It is not mandatory for all team members to be highly experienced, since developing research capacity is also important, however all team members should have defined roles.
Patient and public engagement	Depending on the funding body it may be very important to thoroughly consider patient and public involvement in the study design, development of the research aim planning of the study design, written grant proposal and participation in the proposed study [22]. Engaging the public in the research can improve the quality and impact of the research proposal [23].
Ethics and governance	Details of ethics board approvals including to be obtained for the study are crucial as are details of all governance measures followed.

### Proposed budget

The budget should be designed based on the needs of the project and the funding agency’s policies and instructions. Each aspect of the budget must be sufficiently justified to ensure accountability to the grant awarding body [35]. Costing and justification of the time of those involved, any equipment, consumables, travel, payment for participants, dissemination costs and other relevant costs are required. The funders will be looking for value for money and not necessarily a low-cost study. Ensure that the total budget is within the allocated funding frame.

### Timeline

Provide a breakdown of the key work packages and tasks to be completed, as well as an indication of the anticipated duration. Include a Gantt chart (A table detailing the most general project content milestones and activities) to demonstrate that all aspects of the proposal have been well thought through [21].

### Critical appraisal, limitations, and impact of the proposed research

It is important to detail any strengths and limitations of the proposed project. Omitting these will present the reviewing board with sufficient grounds to reject the proposal [36]. Provide a clear statement about the short and long-term impact of the research [37, 38]. The reviewers will pay particular attention to the differences the study can make and how potential impact aligns with the funding bodies goals as well as national policies. This statement is essential to make an informed decision whether or not to support the application. Useful diagrams summarise the different levels of impact [39].

Table 2 provides a summary of the key elements of project grants and key questions to ask oneself.

**Table 2:** Summary of the key elements of project grants and key questions to ask oneself. (Adapted from [5]: Koppelmann GH, Holloway JW. Successful grant writing. *Paediatr Respir Rev.* 2012; 13:63–66.)

Key questions to ask oneself	
What is the research question being addressed? How important, or how big is the identified knowledge gap? Why is this research project needed? What previous literature is available on this research topic? How innovative is the grant proposal compared to already published or ongoing research? What would the impact of the study results on healthcare, economics and society be? What research is being done by other groups? What type of methodological approach would be required in an ideal world to address this issue? What is needed to bring this research project to a wider audience? Does the researcher and team have all the relevant skills, techniques, and knowledge? Am I ready to be a principal investigator or should I be a co-investigator?	

## Conclusion

Although the grant writing process is time-consuming and complex, support is widely available at each stage. It is important to involve colleagues and collaborators to improve the proposal as much as possible and invest time in the detailed planning and execution. Even if the grant is not awarded, do not be disheartened. Use the feedback for improvement and exercise resilience and persistence in pursuing your research ambition.

The guidance in this paper is part of ESCP’s commitment to stimulate “innovative and high-quality research in all areas of clinical pharmacy”. In a previous ESCP survey, it was found that few opportunities for collaboration (especially for grant applications) was one of the key barriers for members towards conducting research [40]. ESCP promotes networking, which is essential for multi-centre grant applications, both among ESCP members and with other organisations as it recognises the need for “multi-centre research in all areas of clinical pharmacy both within countries and between countries or differing healthcare delivery systems”. ESCP is planning to relaunch its own research grant which was paused during the pandemic, and it is also planning to provide ESCP members with information about the research grants offered by other organizations. ESCP is exploring partnering with other organisations to develop research proposals in areas of common interest and, in the near future, it will ask its members about their research priorities. Taken together, these initiatives will inform ESCP’s research strategy and help it to formulate policies to address the challenges its members face.
